# Ruptured primary ovarian pregnancy: A case report with a literature review

**DOI:** 10.1097/MD.0000000000039023

**Published:** 2024-07-19

**Authors:** Xuemei Qing, Min Xie, Yong Zhang, Ying Ma

**Affiliations:** a Department of Obstetrics and Gynecology, Qingbaijiang District People’s Hospital, Chengdu, Sichuan, China; b Department of Obstetrics and Gynecology, Mianyang Central Hospital, Mianyang, Sichuan, China.

**Keywords:** case report, laparoscopy, ovarian pregnancy, rupture, surgery

## Abstract

**Rationale:**

Ovarian pregnancy is a rare form of ectopic pregnancy, accounting for 0.5% to 3% of the total number of ectopic pregnancies. Its diagnostic rate is very low and it can be easily misdiagnosed before laparoscopy, due to the clinical presentation being very similar to tubal pregnancy. The ovarian blood supply is abundant, and in case of rupture of ovarian pregnancy, intra-abdominal hemorrhage or even hemorrhagic shock may occur, endangering the patient’s life. We report a case of ruptured primary ovarian pregnancy through natural conception.

**Patient concerns:**

This patient had a history of menopause with lower abdominal pain and tenderness. Ultrasound showed a thick-walled cystic echo in the left adnexal region, a dark area of fluid in the pelvis, and an irregular, slightly strong echo posterior to the uterus. Unclotted blood was punctured from the posterior fornix, and her hemoglobin was decreasing with a serum β-human chorionic gonadotropin of 1800.00 mIU/mL.

**Diagnoses:**

Through early recognition of clinical manifestations, ultrasonography, laparoscopic exploration, and the final histopathologic examination, this patient was diagnosed with an ovarian pregnancy.

**Interventions:**

Then, removal of the left ovarian pregnancy lesion was performed, which was visible as villi. And Methotrexate 50 mg was administered locally.

**Outcomes:**

Through conservative surgical treatment, she recovered well and was discharged with a satisfactory follow-up.

**Lessons:**

Gynecologists should be alert to patients with menopausal lower abdominal pain with or without vaginal bleeding and consider ectopic pregnancy in rare sites, such as ovarian pregnancy. Surgery is the mainstay of treatment, and early laparoscopic exploration may be beneficial in clarifying the diagnosis and performing the concurrent surgical treatment.

## 1. Introduction

Ectopic pregnancy is a condition in which the fertilized ovum is deposited and develops outside the uterine cavity with a prevalence of only 1% to 2%, but the mortality rate in early pregnancy is as high as 75%, occurring mainly in the fallopian tubes and rarely in the uterine horns, cervix, ovary or abdominal cavity. Ovarian pregnancy (OP) is a rare form of ectopic pregnancy, accounting for 0.5% to 3% of all ectopic pregnancies. Its diagnostic rate is very low and it can be easily misdiagnosed before laparoscopy, due to the clinical presentation being very similar to tubal pregnancy.^[1]^ Due to the rich blood supply of the ovary, intra-abdominal hemorrhage or even hemorrhagic shock may occur, once the ovarian pregnancy ruptures. Therefore, early recognition of clinical manifestations, timely laparoscopic confirmation of diagnosis, and simultaneous surgical treatment have become the key to the management. Here, we report a rare case of a ruptured primary ovarian pregnancy that was diagnosed and operated on laparoscopically with final pathologic confirmation. Informed consent was obtained from the patient, and this article is a case report that does not require ethical approval.

## 2. Case presentation

The patient, a 42-year-old female, was admitted to the hospital on January 19, 2024, with “menopause for 34 days and abdominal pain for more than 5 hours.” She had a menstrual cycle of 23 to 27 days with a normal menstrual cycle of 4 days, a history of G_3_P_1_^+2^, a live birth by cesarean section in 2006, and 2 previous miscarriages. Her last menstrual period was on December 16, 2023, and she had no menopausal nausea or vomiting. More than 5 hours earlier, she had experienced lower abdominal cramping that was initially tolerable and then worsened slightly with nausea and no other discomfort. She came to our hospital for an ultrasound examination, which revealed a left adnexal mass, and was subsequently evaluated as having an ectopic pregnancy and admitted to the hospital for further management. The patient had no history of chronic or infectious diseases or drug allergies.

Physical examination revealed a temperature of 36.2 °C, pulse rate of 72/min, blood pressure of 108/61 mm Hg, pressure and rebound pain in the lower abdomen, but no obvious muscle tension, and other signs were negative. Gynecological examination: normal vulvar development, married and unproductive type, patent vagina with white discharge, smooth cervical surface, normal uterus size, good mobility with pressure and pain, thickening of the left adnexal area with pressure and pain, and no palpable mass or pressure in the right adnexal area.

Ancillary examination transvaginal ultrasound: the uterus is in a horizontal position with normal morphology, anterior–posterior diameter of about 4.4 cm, myometrium with uniform echogenicity, homogeneous endometrial bilayer with thickness of about 0.9 cm, right adnexal region un-clear, and thick-walled cystic echoes approximately 2.8 × 2.7 cm in the left adnexal region. Abdominal ultrasound: a fluid dark area with a depth of about 3.8 cm in the pelvic cavity; an irregular slightly strong echogenicity of about 5.8 * 1.4 cm behind the uterus. Urine ultrasonography: no abnormality. Approximately 3 mL of non-clotted blood was punctured from the posterior fornix. Hemoglobin (HGB) was 103 g/L and serum beta-human chorionic gonadotropin (β-HCG) was 1800.00 mIU/mL.

**Figure 1.**
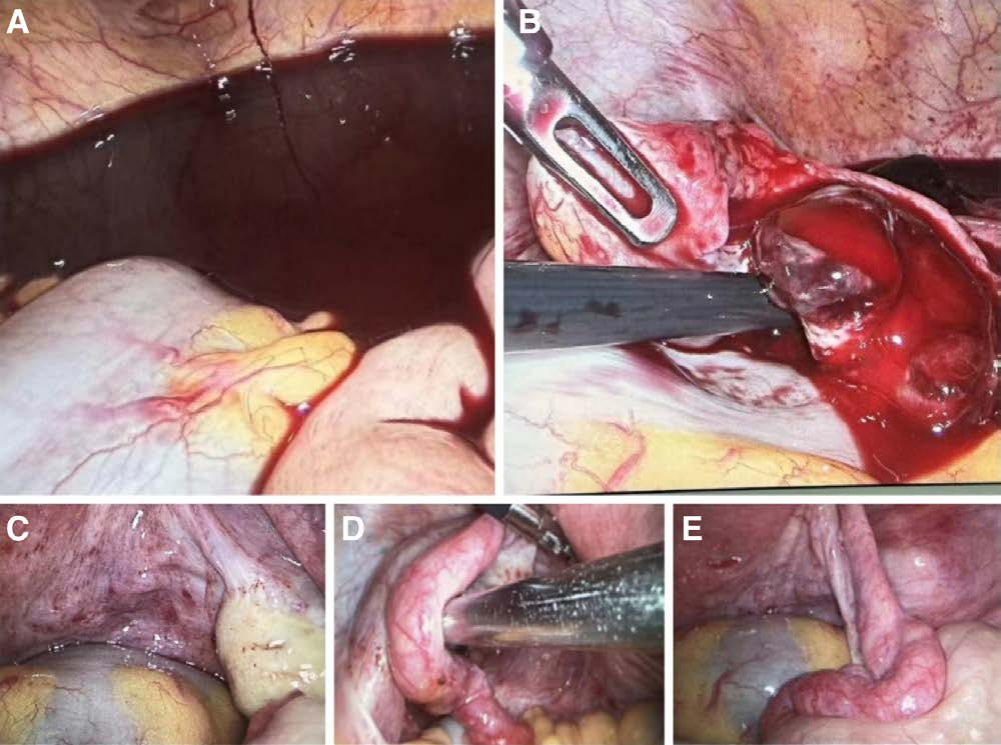
Laparoscopic views. (A) The accumulation of free blood and clots in the pelvic and abdominal cavity; (B) the left ruptured ovarian pregnancy; (C) the right ovary with normal appearance; (D) the left fallopian tube with normal appearance; (E) the right fallopian tube with normal appearance.

**Figure 2.**
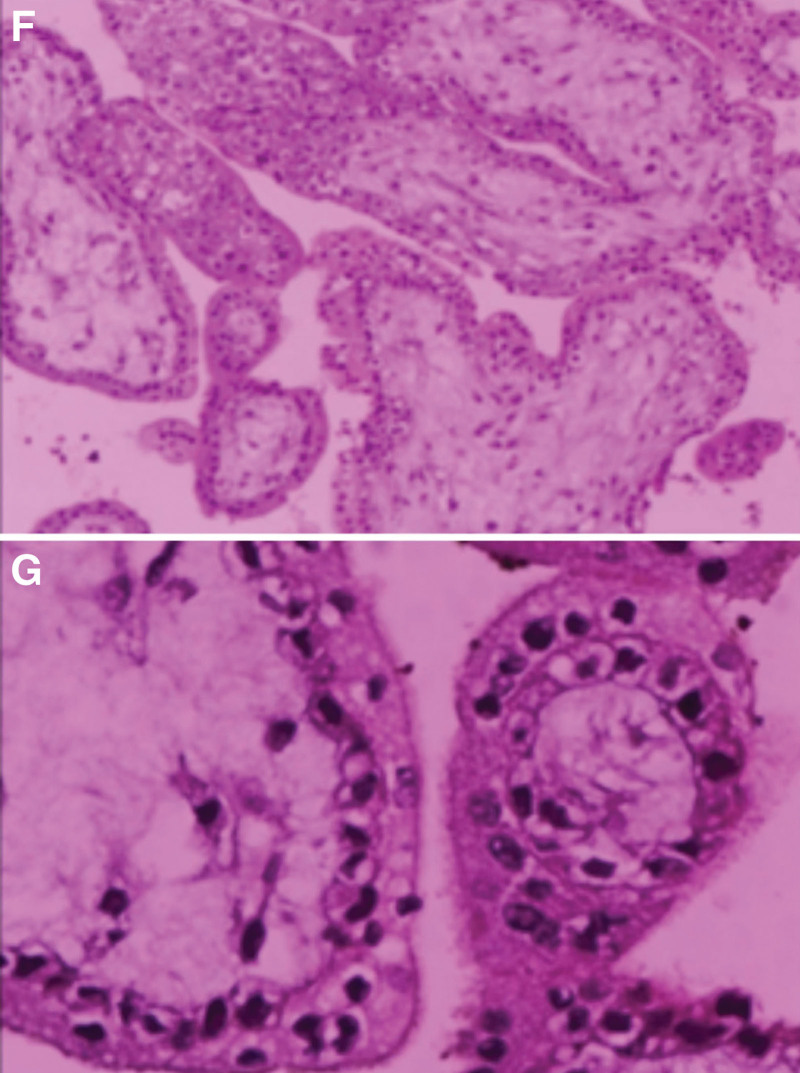
Pathology of focal tissue within the left ovary, H&E staining (F: ×10; G: ×40). Microscopically, the tissue shows a large amount of chorionic tissue, consistent with pregnancy.

**Figure 3.**
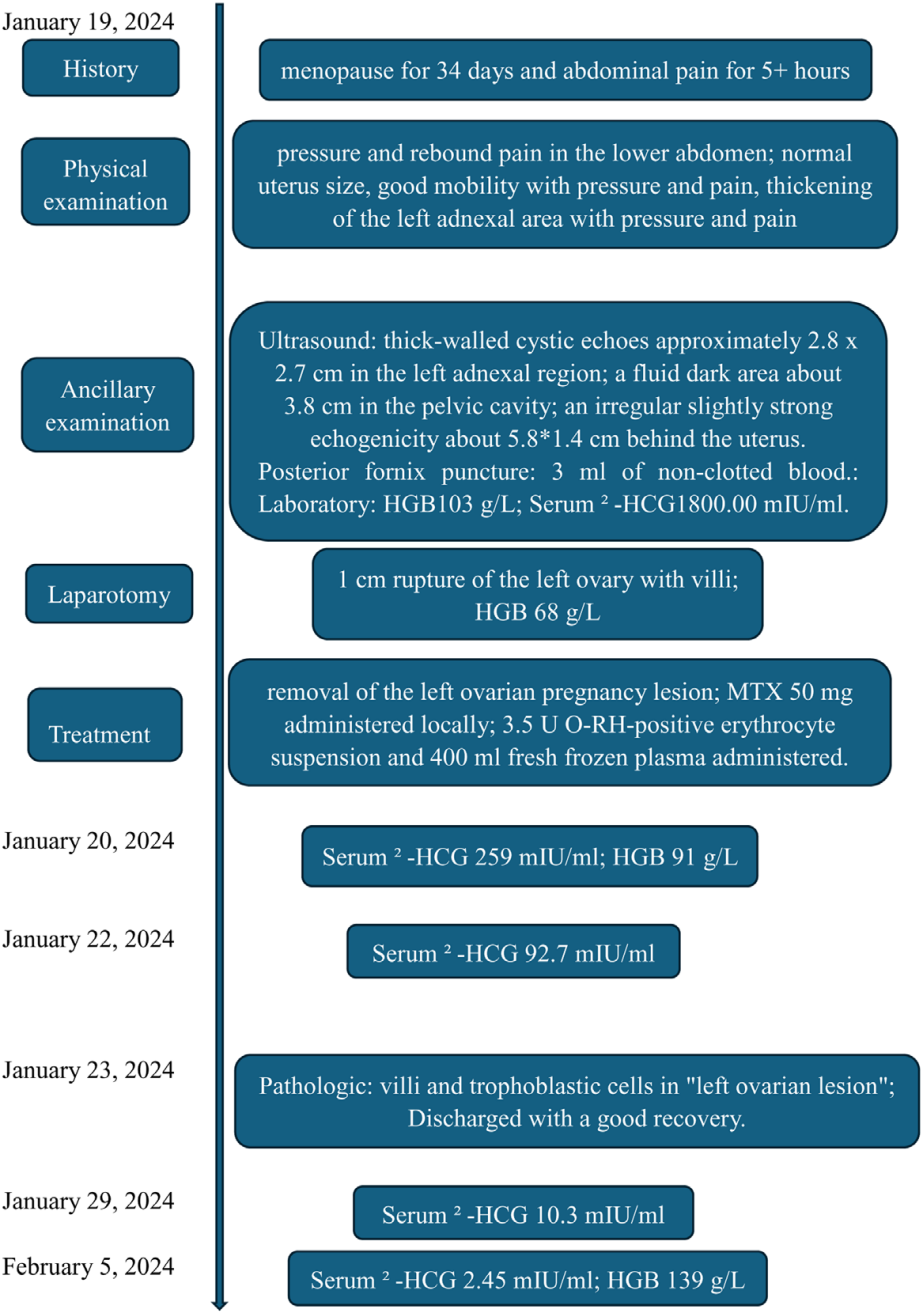
Timeline.

Together with the history, physical examination, and ancillary tests, the diagnosis was: ectopic pregnancy? intra-abdominal hemorrhage; and mild anemia. We performed an emergency exploratory laparotomy (Fig. 1). The accumulation of residual blood and clots intraoperatively was approximately 1400 mL, and a 1 cm rupture of the left ovary with villi, corpus luteum, and blood clots, with active bleeding. The bilateral fallopian tubes and right ovary were with normal appearance. Then, removal of the left ovarian pregnancy lesion was performed, which was visible as villi. Methotrexate (MTX) 50 mg was administered locally. A total of 3.5 U O-RH-positive erythrocyte suspension and 400 mL fresh frozen plasma were administered for the HGB, which was 68 g/L intraoperatively and increased to 91 g/L after transfusion. On the first postoperative day, the serum β-HCG level decreased to 259 mIU/mL, and on the third day it decreased to 92. 7 mIU/mL. Pathologically, there were villi in the “left ovarian lesion” (Fig. 2). The patient was discharged with a good recovery on the fourth day. Subsequently, she was followed up in outpatient: the serum β-HCG level decreased to 10.3 mIU/mL on the 10th day, and to the normal level with a result of 2.45 mIU/mL on the 17th day. At the same time, the HGB increased to 139 g/L, and the liver function was also at the normal level. The timeline is detailed in Figure 3.

## 3. Discussion

OP is a pregnancy process in which a fertilized ovum is deposited and develops in the ovary, with an incidence of 1/7000 to 1/50,000,^[2]^ which may have increased in recent years due to the use of intrauterine devices and the spread of assisted reproductive technology.^[3]^ Most ovarian pregnancies rupture early, resulting in intra-abdominal hemorrhage or even shock, which is very similar to the clinical presentation of tubal pregnancy. Ovarian pregnancy is often difficult to diagnose or easily misdiagnosed, and laparoscopy is often necessary to confirm the diagnosis. This patient is considered to have an ectopic pregnancy, but the location is unknown, then laparoscopic exploration clarifies the pregnancy lesion in the left ovary and rupture.

Depending on the location of the fertilized ovum in the ovary, ovarian pregnancy can be classified as primary or secondary OP. The most common clinical primary ovarian pregnancy, i.e. intrafollicular type, refers to the failure of follicular ovulation, the fertilized ovum is deposited in the early corpus luteum and develops in the ovary where the ovarian tissues completely encapsulate the embryo. Secondary OP is when the fertilized embryo reaches the ovary by retrograde peristalsis through the fallopian tube and develops on the surface of the ovary, where the ovarian tissue may be part of the gestational sac wall.^[4]^

### 3.1. Pathogenesis

The exact etiology of ovarian pregnancy is unknown. It may be associated with pelvic inflammatory disease, endometriosis, intrauterine device placement, assisted reproductive technology, and previous pelvic surgery with the following pathogenesis.

### 3.2. Ovulatory disorders

Polycystic ovary syndrome is a common ovulatory disorder in which hyperandrogenism prevents the formation of dominant follicles and ovulation, increasing the likelihood of intrafollicular fertilization.^[5]^ Previous pelvic surgery or ovarian inflammation can lead to thickening and adhesions in the ovarian cortex, which reduces intrafollicular pressure and prevents ovulation, and may also lead to intrafollicular fertilization.^[6]^ Endometriosis causes the surface of the ovary to metaplasia for fertilization of the ovum, and OP may occur if there are endometriotic lesions on the ovary.^[7]^

### 3.3. Tubal dysfunction

Inflammation of the fallopian tubes or previous pelvic surgery can lead to thickening of the tubal wall, fluid accumulation in the lumen, abnormal movement of the endothelial cilia, and increased retrograde peristalsis, which can lead to abnormal transport of the fertilized ovum. In the presence of tubal adhesions, the fertilized ovum may not be properly transported and deposited in the uterus, resulting in deposition on the surface of the ovary, leading to OP.^[6]^ In addition, intrauterine devices can induce retrograde tubal peristalsis, leading to secondary OP.^[8]^ Previous cesarean section in this patient is a risk factor for the development of OP.

### 3.4. In vitro fertilization-embryo transfer

Since the first report of ovarian and contralateral tubal pregnancy after assisted reproductive technology, the incidence, and reporting of OP have increased.^[9]^ In addition, the use of ovulation-inducing drugs and ovarian stimulation increases uterine contractility and embryo sac migration to the fallopian tube and ovary.^[10]^

## 4. Diagnosis

### 4.1. Clinical manifestations

Menopausal abdominal pain and/or vaginal bleeding is common, with or without anal swelling. As a result, ovarian pregnancy can be easily misdiagnosed and the diagnostic rate is low, often requiring surgical exploration to clarify the diagnosis. Most ovarian pregnancies rupture early, resulting in intra-abdominal hemorrhage and even shock. These clinical manifestations are extremely similar to those of tubal pregnancy. Approximately 5.3% of patients with ovarian pregnancies will continue into the second trimester, 3.7% will continue into late pregnancy, and a very small percentage of patients may carry the pregnancy to term or even live birth.^[11]^ Therefore, diagnosing ovarian pregnancy based on clinical presentation alone is limited.

### 4.2. Ultrasonography

The ultrasonographic features of an ovarian pregnancy are: the absence of a gestational sac in the uterus; an adnexal mass that is cystic or embryonic in shape; and a thick and loose wall of the sac (ovarian tissue). Based on ultrasound, OP can be classified as ruptured or unruptured, with the latter subdivided into cystic masses and inhomogeneous masses. Early diagnosis by transvaginal ultrasound is beneficial in terms of operative risk and patient life.^[12]^ This patient’s ultrasound reminds us gynecologists to pay enough attention so we can treat aggressively. The above ultrasound features can be obtained only when the ovarian pregnancy has not ruptured. However, in most clinical practices, patients come to the hospital due to worsened symptoms, maybe the ovarian pregnancy has already ruptured, with a mixed echogenic mass in the adnexal area and pelvic blood or blood clots, like this patient, which makes it difficult to distinguish it from a tubal pregnancy. Pelvic computed tomography or magnetic resonance imaging is rarely used to diagnose. Therefore, a clear diagnosis needs laparoscopic exploration.

### 4.3. Laparoscopy

Laparoscopy is the gold standard for diagnosis of ovarian pregnancy and can be used to identify the site of pregnancy and bleeding and guide appropriate surgical management.^[1]^ This patient is definitively diagnosed through laparoscopy. It also has the advantage of surgical treatment, which is determined by the type of ovarian pregnancy, whether it is ruptured, the severity of ovarian destruction, and the amount of intra-abdominal bleeding. Although open exploration is also a means of evaluation and treatment, it has been gradually replaced by minimally invasive laparoscopic surgery due to its large incision, slow recovery, and increased likelihood of pelvic adhesions in the distant future.

### 4.4. Pathologic diagnosis

The diagnosis of ovarian pregnancy depends mainly on the pathologic findings, and the diagnostic criteria proposed by Spiegelberg, which have been widely used since 1878, include the following 4 criteria: normal fallopian tubes bilaterally; follicles located within the ovarian tissue; follicles and ovaries connected to the uterus by the ovarian ligaments; and follicular walls with ovarian tissue. However, in clinical practice, all 4 criteria are not always met simultaneously. This patient met the first 3 criteria, but not the fourth, because the pathologic examination did not reveal any ovarian tissue in the follicular wall, which may have been due to the intraoperative exploration that confirmed a ruptured pregnancy in the left ovary, and only the pregnancy lesion was removed.

## 5. Treatment

### 5.1. Surgery

Surgery is the mainstay of treatment for OP. Laparoscopic surgery is the ideal choice, allowing for local magnification and definitive diagnosis, as well as appropriate surgery characterized by minimal trauma and rapid recovery. Depending on the size of the lesion, ovarian pregnancy lesion removal, ovarian wedge resection, or partial oophorectomy may be performed. Ovarian tissue is preserved as much as possible. In cases of severe ovarian destruction, adnexectomy of the affected side may be performed.^[13]^ A simple removal of the ovary with preservation of the ipsilateral fallopian tube is generally not recommended to prevent displacement of the fertilized ovum and tubal pregnancy. The recurrence rate of ovarian pregnancy is very low, the pregnancy rate after treatment is high, and the impact on female fertility is low.^[14]^ Removing the ovarian pregnancy lesion is appropriate in this patient, as the serum β-HCG drop is very satisfactory postoperatively.

### 5.2. Medication

The most commonly used medication is methotrexate, which can be injected into the gestational sac under ultrasound guidance in unruptured ovarian pregnancies. If laparoscopy confirms an unruptured ovarian pregnancy, we can still inject MTX locally. Of course, systemic injection of MTX is also possible. The effect of MTX on intrauterine pregnancy is controversial because of its potential embryotoxicity. However, recent studies have shown that local injection of minute amounts of MTX is safe and effective, and there is no evidence of adverse effects of MTX on ovarian reserve function.^[1]^

In this case, ectopic pregnancy was suspected based on clinical presentation and ultrasound, laparoscopy was performed due to uncertainty of the site, exploration revealed a follicle at the rupture of the left ovary, which was confirmed as a ruptured ovarian pregnancy, and removal of the left ovarian pregnancy lesion was performed. Pathologic examination revealed chorionic villi and satisfactory monitoring of serum β-HCG decline after surgery supported the appropriateness of the procedure. Ovarian pregnancy is prone to early rupture, causing hemorrhage or even shock, with a higher risk than tubal pregnancy due to its rich blood supply. Therefore, it should be treated as early as possible after detection, and the combination of ultrasound and laparoscopic diagnosis with surgery and pathological examination can achieve the best therapeutic outcome.

## Author contributions

**Conceptualization:** Xuemei Qing.

**Data curation:** Xuemei Qing, Min Xie.

**Supervision:** Xuemei Qing, Yong Zhang, Ying Ma.

**Validation:** Xuemei Qing, Ying Ma.

**Writing – original draft:** Xuemei Qing.

**Writing – review & editing:** Xuemei Qing, Min Xie, Yong Zhang, Ying Ma.
